# Electric discharge during electrosurgery

**DOI:** 10.1038/srep09946

**Published:** 2015-04-16

**Authors:** Alexey Shashurin, David Scott, Taisen Zhuang, Jerome Canady, Isak I. Beilis, Michael Keidar

**Affiliations:** 1 Department of Mechanical and Aerospace Engineering, School of Engineering and Applied Science, The George Washington University, Washington, DC 20052, USA; 2 Jerome Canady Research Institute for Advanced Biological and Technological Sciences, 6930 Carroll Avenue, Suite 300, Takoma Park, MD 20912; 3 School of Electrical Engineering, Tel Aviv University, Ramat Aviv 69978

## Abstract

Electric discharge utilized for electrosurgery is studied by means of a recently developed method for the diagnostics of small-size atmospheric plasma objects based on Rayleigh scattering of microwaves on the plasma volume. Evolution of the plasma parameters in the near-electrode sheaths and in the positive column is measured and analyzed. It is found that the electrosurgical system produces a glow discharge of alternating current with strongly contracted positive column with current densities reaching 10^3^ A/cm^2^. The plasma electron density and electrical conductivities in the channel were found be 10^16^ cm^−3^ and (1-2) Ohm^−1^cm^−1^, respectively. The discharge interrupts every instance when the discharge-driving AC voltage crosses zero and re-ignites again every next half-wave at the moment when the instant voltage exceeds the breakdown threshold.

Electrosurgery has been utilized for cutting and coagulating tissue for about 90 years^1^,^2^. Electrosurgical coagulation has improved treatment of many gastrointestinal diseases such as radiation proctitis, Barrett’s esophagus, gastric antral vascular ectasia, and arteriovenous malformations^3^,^4^,^5^,^6^,^7^,^8^,^9^,^10^. Additionally, it may decrease postoperative swelling and inter-operative blood loss for other areas of the human body such as knee joint replacement.

Electrosurgical Argon Plasma Coagulation (APC) utilizes plasma produced by the ionization of a few millimeter diameter argon flow exhausting into ambient air from the electrosurgical hand-piece. The intensity of treatment and the effect induced in the living tissue strongly depends on the plasma properties^11^. Such atmospheric pressure microplasmas are difficult to study using conventional diagnostics. Microwave interferometry operating in the GHz frequency range fails due to the small size of the plasma compared to the microwave wavelength causing diffraction and unsufficient phase change. Electrostatic probes introduce very strong perturbations once inserted into the plasmas, and are associated with difficulties of interpretation at strongly-collisional atmospheric conditions^12^,^13^.

Recently a few methods for the measurement of atmospheric pressure plasma parameters were proposed. This includes various spectroscopic techniques, namely passive optical emission spectroscopy, laser-induced fluorescence spectroscopy, diode laser absorption spectroscopy and Rayleigh, Thomson and Raman scattering of laser radiation on microplasmas^14^,^15^,^16^,^17^,^18^,^19^,^20^,^21^. These techniques are characterized by good spatial resolution (down to 10–50 μm) and minimal detectable values of plasma electron density of ~10^13^ cm^−3^, but require great stability of the discharge since they are based on averaging over large number of discharge events.

An alternative approach capable of detecting low plasma densities (down to 10^11^–10^12^ cm^−3^) at atmospheric pressure is Rayleigh scattering of microwave radiation on microplasmas^22^. The concept of the method was first proposed theoretically by Shneider^23^, and then implemented experimentally in studies of laser-induced avalanche ionization in air, resonance-enhanced multi-photon ionization in argon and non-thermal atmospheric plasma jets (widely utilized recently for biomedical applications)^22^,^24^,^25^,^26^,^27^,^28^. The method consists of measurements of radiation scattered from microplasmas irradiated by microwaves in the GHz frequency range. In the Rayleigh regime, the electric field amplitude of the scattered wave is proportional to total number of electrons in the microplasmas and thus, the plasma electron density can be determined if the plasma volume is known^23^.

The type of discharge and the plasma parameters utilized during electrosurgical coagulation has not been extensively studied, even though this type of plasma source probably has the widest practical application in medicine thus far. In this work, we utilize an electrosurgical system produced by US Medical Innovations LLC, and present for the first time, measurements of plasma discharge parameters and discuss processes developing in the positive column and in the near-electrode sheath.

## Experiment

### Electrosurgical system

**Figure 1:**
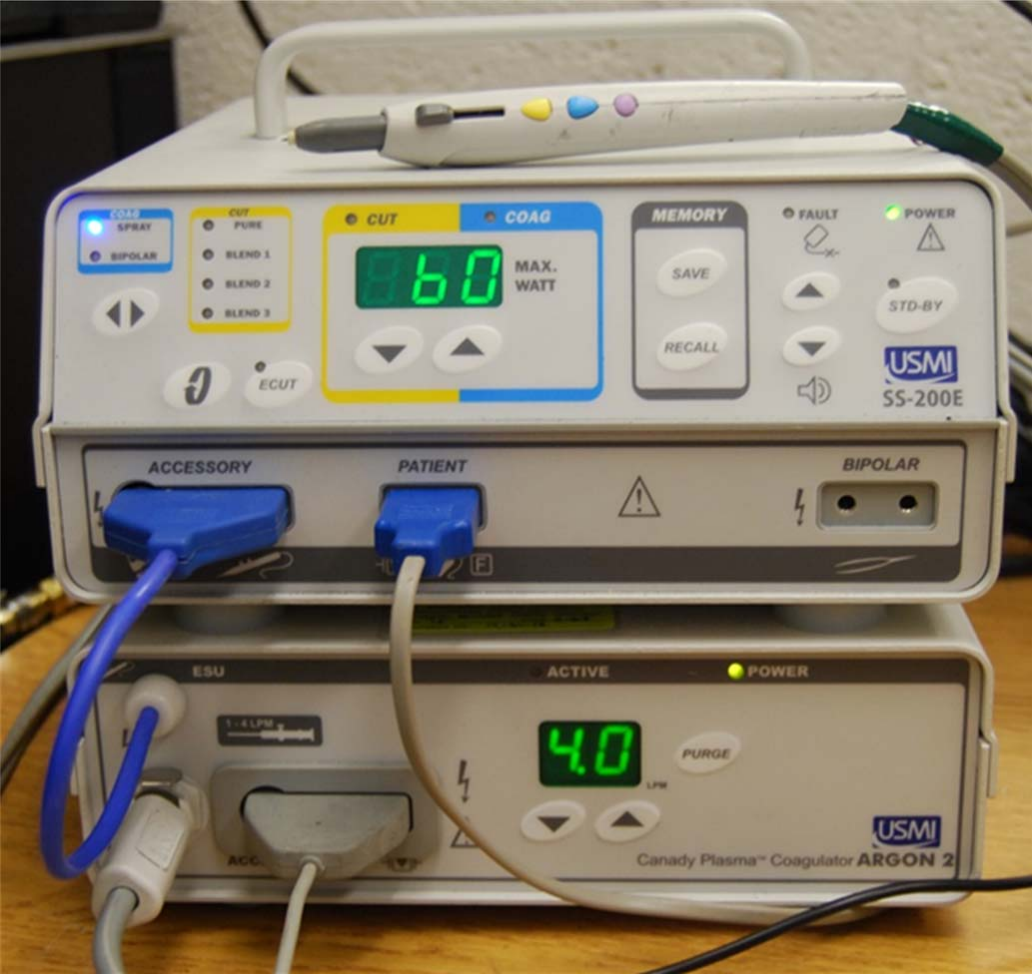
Photograph of electrosurgical system SS-200E/Argon 2 with connected electrosurgical Canady Vieira Hybrid Plasma Scalpel by US Medical Innovations, LLC.

The experiments were conducted using the electrosurgical system SS-200E/Argon 2 in combination with the electrosurgical Canady Vieira Hybrid Plasma scalpel by US Medical Innovations LLC shown in Fig. 1 (see Ref. [29] for histological studies). The Canady Vieira Hybrid Plasma scalpel is comprised of a flexible hose ending with a hand-piece^29^. The hose delivers argon flow and electrical power produced by the electrosurgical generator to the hand-piece. The hand-piece is nearly a cylindrical hollow volume with a high-voltage tungsten electrode of about 1 mm in diameter installed on the axis and surrounded by the argon flow. The hand-piece is equipped with a 2.4 mm opening at its distal end through which the argon flow exits into the ambient air. The electrode was protruded about 5 mm from the distal end in the experiments. The electrosurgical unit (ESU) was utilized in a range of power settings from 15 to 60 Watt and 3 LPM argon flow rate.

### Inorganic replica of the living tissue

**Figure 2:**
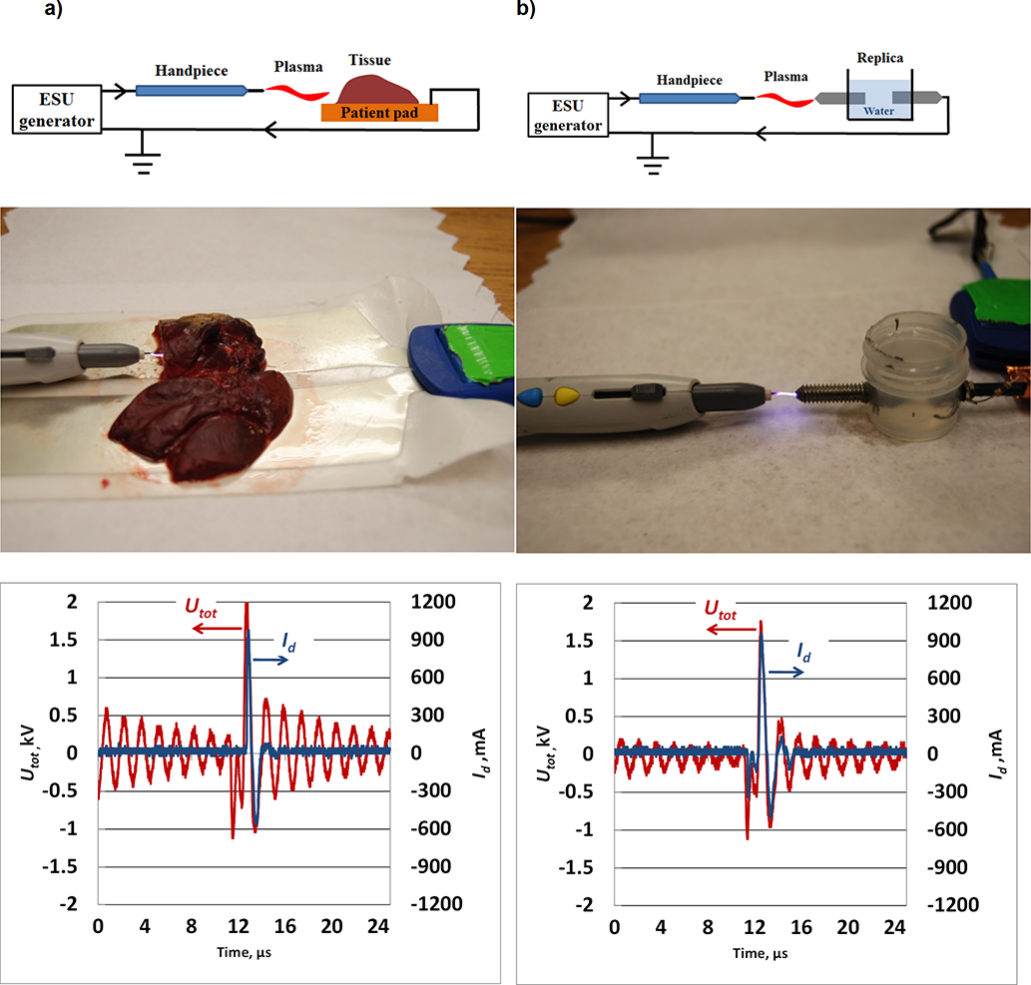
Photograph of the ESU in operation in Argon Plasma Coagulation mode, electrical schematics and current/voltage waveforms of the discharge for ESU power 60 W and flow rate - 3-3.5 LPM: (a) chicken liver sample, (b) inorganic replica. Similar voltage and current waveforms indicate good agreement of the electrical properties of tissue sample and inorganic replica.

The power produced by the ESU is delivered into the biological tissue by means of an argon plasma column producing an electrically conductive channel between the high voltage electrode of the electrosurgical hand-piece and the tissue. A photograph of the SS-200E/Argon 2 in operation in Argon Plasma Coagulation (APC) mode with a fresh chicken liver sample is shown in Fig. 2(a). Application of the ESU to the tissue sample produces instant tissue burning, accompanied with smoke production at the treated point [see Fig. 2(a)]. Additionally, the plasma column attachment to the tissue is non-steady and characterized by fluctuations and motion of the plasma column around the tissue.

To avoid problems with the non-reproducibility of the measurements, due to non-steady motion of the plasma column over the tissue, the experiments were conducted using an inorganic replica of the tissue made of two metal electrodes separated by water gap [see insert in Fig. 2 (b)]. A photograph of the ESU in operation in the APC mode with the inorganic replica is shown in Fig. 2 (b). It was observed that the discharge with the inorganic replica sample produced quasi-steady, immovable diffusive attachment to the replica electrode tip as shown Fig. 2 (b). All measurements were conducted at a separation distance between the replica electrode tip and the electrosurgical hand-piece tip of about *d* = 4.5 mm.

The water gap size in the replica sample was adjusted to obtain current and voltage waveforms similar to that of the chicken liver tissue samples. The waveforms of the voltage produced by the ESU (*U_tot_*) and the discharge current (*I_d_*) were similar for both samples (peak voltage of about 2 kV and peak current ~1 A for 60 W and 3-3.5 LPM) as shown in Fig. 2.

The details of the electrical schematics are shown in Fig. 2. The electrical circuit was as follows: ESU (Accessory port) → electrosurgical scalpel → tissue on patient pad/replica → ESU (Patient port). Note, for experiments conducted with biological samples tissue was placed on the standard patient pad, while inorganic replica experiments were made with no patient pad (see schematic in Fig. 2). The Patient port of the ESU was grounded in all experiments as shown in Fig. 2.

### Microwave scattering diagnostics

**Figure 3:**
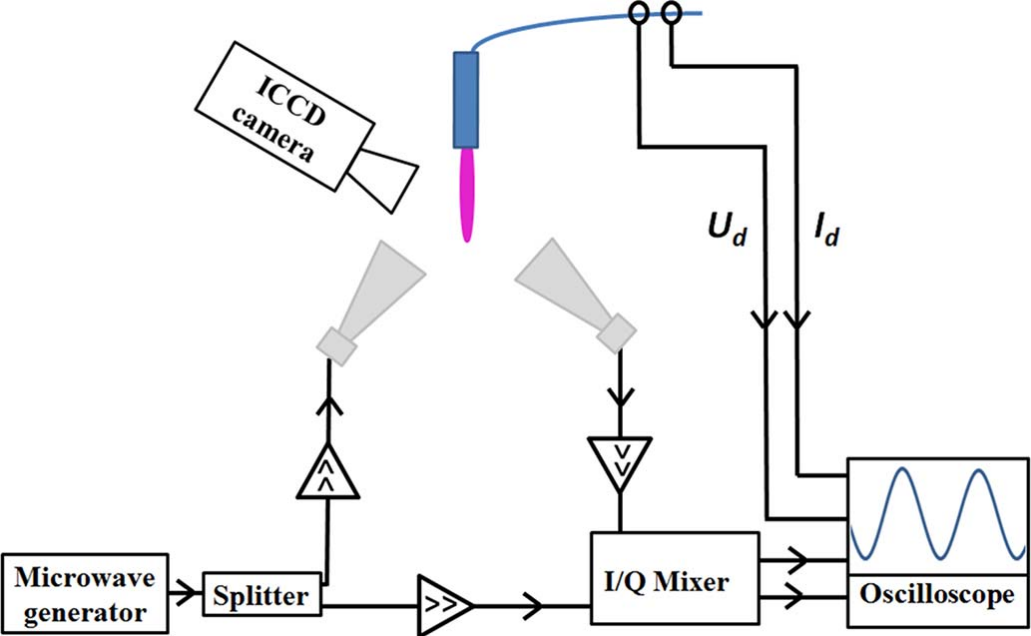
Schematics of the RMS system utilized in this work.

The schematics of the Rayleigh Microwave System (RMS) are presented in Fig. 3. Linearly polarized microwave radiation (10.58 GHz) was scattered on the collinearly-oriented plasma channel and then the scattered signal was measured. Microwaves were irradiated and detected using two horns shown in Fig. 3. The detection of the scattered signal was accomplished using a homodyne I/Q Mixer, providing in-phase (*I*) and quadrature (*Q*) outputs. The total amplitude of the scattered microwave signal was determined by: 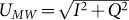. The amplifiers and the mixer used in the microwave system operated in a linear mode for the entire range of the scattered signal amplitudes, thereby ensuring that the output signal *U_MW_* is proportional to the electric field amplitude of scattered radiation *E_s_* at the detection horn location: *U*_*MW*_ = *E*_*S*_.

The absolute value of the plasma electron density, conductivity and electric field in the plasma channel were determined from Eq. (4)–(6) according to the methodology developed in Methods. A digital oscilloscope was used to simultaneously record signals for the microwave system, along with the electrical parameters of the discharge. In addition, the plasma column was simultaneously photographed using an Intensified Charge-Coupled Device (ICCD) camera: Andor USB iStar.

## Results

**Figure 4:**
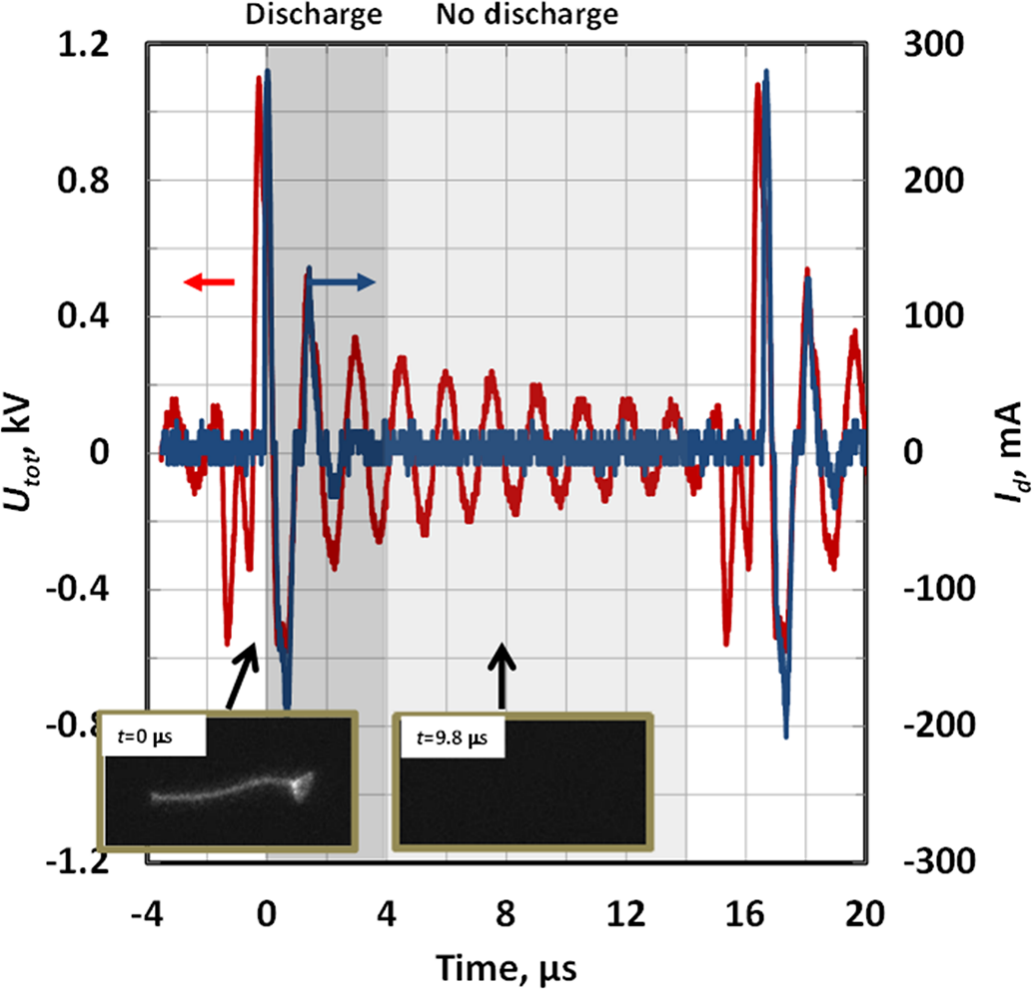
Total AC voltage (*U_tot_*) and discharge current (*I_d_*) produced by the ESU system for an input power of 15 Watts and an argon flow rate of 3 LPM. The discharge-driving voltage represents a sequence of the high frequency, high voltage pulses. The operation cycle has two stages, namely the active (*t*~0–4 μs, darker bar) and inactive stage (*t*~4–16 μs, brighter bar). Typical photographs of each stage are presented for time moments *t* = 0 and 9.8 μs respectively (exposure time 20 ns).

Fig. 4 presents the waveforms of the total voltage produced by the ESU (*U_tot_*) as well as the discharge current (*I_d_*). One can see that the ESU produces a series of high voltage bursts, having a peak amplitude of about 1 kV and a repetition frequency of about 60 kHz which are filled with sinusoidal oscillations at about 600 kHz. Two stages can be distinguished on the operation cycle, namely the active and inactive stage as indicated on the Fig. 4 by the darker and brighter bars, respectively. The active stage (*t*~0-4 μs) is characterized by the presence of the non-zero discharge current spikes (*I_d_* peak values up to 250-300 mA) and a bright plasma column oscillating between the discharge electrodes as shown in the typical discharge image in Fig. 4 (the image is taken at *t* = 0 μs). The inactive stage (*t*~4–16 μs), is characterized by an absence of the discharge (*I_d_* = 0) and a dark inter-electrode gap.

**Figure 5:**
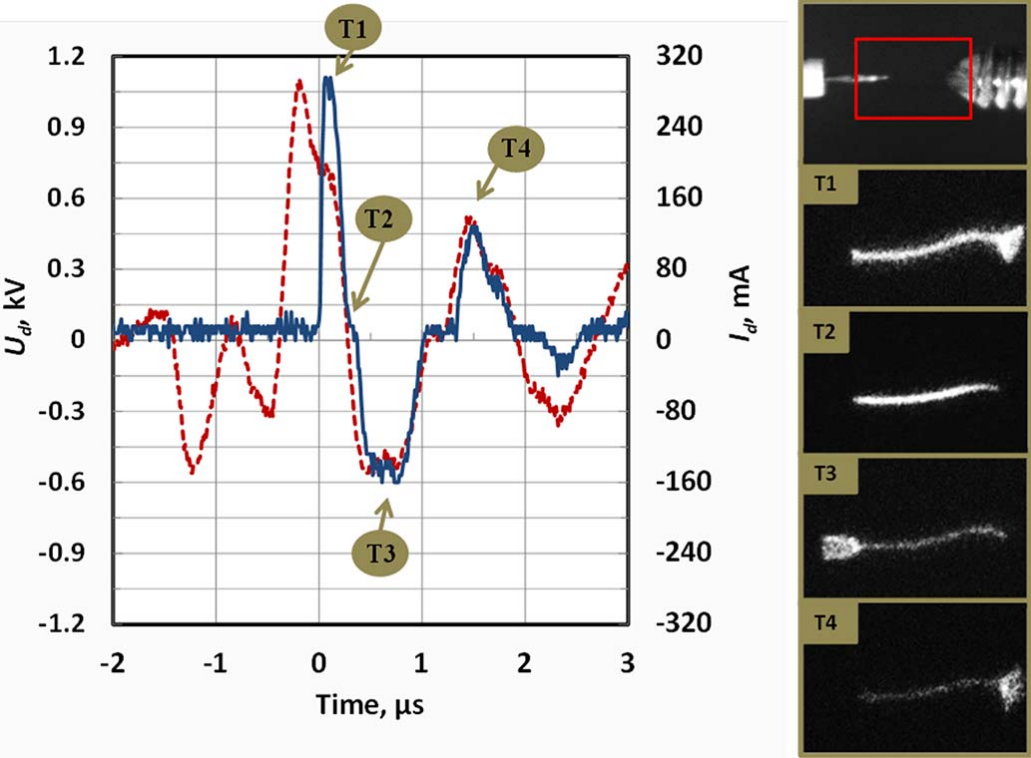
Temporal evolution of discharge voltage (*U_d_*) and discharge current (*I_d_*) of the electrosurgical system SS-200E/Argon 2 for *P* = 15W and flow = 3 LPM. Photographs of the interelectrode gap at different moments of time T1-T4 are shown on the right.

The active discharge stage is shown in more details in Fig. 5. It was observed that a bright discharge column develops between the electrodes in the volume occupied by argon gas and oscillates overlapping (covering) left and right discharge electrodes by turns (see e.g. image *T1* in Fig. 5 where plasma column covers the tip of the right electrode; image *T3* – same for the left electrode). One can see that the plasma column always overlaps the electrode that is negative at this particular moment (instant cathode) - see images *T1*, *T3* and *T4* in Fig. 5. Time intervals in vicinity of the voltage zeroes (*U_tot_*~0) were associated with the presence of the plasma column strictly between the electrodes without any electrode overlapping - see image *T2* in Fig. 5.

**Figure 6:**
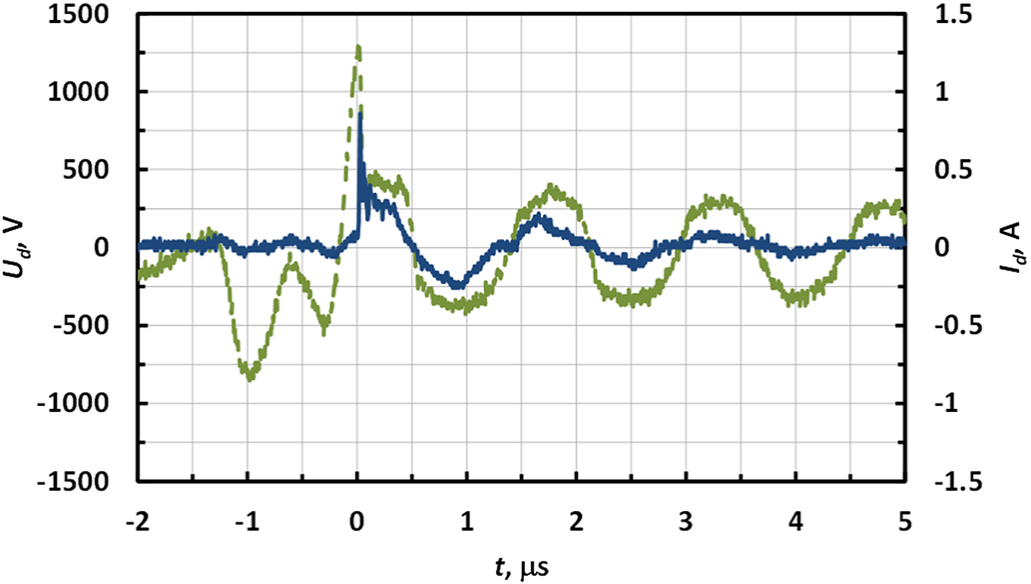
Discharge voltage (*U_d_*) and discharge current (*I_d_*) for 60 Watts and argon flow rate 3 LPM.

The total AC voltage produced by the ESU (*U_tot_*) represents the sum of the discharge voltage (*U_d_*) and the voltage drop on the replica/tissue sample (*U_sample_*): *U_tot_* = *U_d_+U_sample_*. *U_d_* was determined by subtracting the voltage measured at the surgical probe from that measured at the replica electrode. Waveforms of the discharge voltage and current are shown in Fig. 6. One can see that breakdown occurred at about 1.2 kV and afterwards the discharge voltage was generally in the range ~300–400 V.

The diameter and length of the interelectrode plasma channel and the area of its attachment to the discharge electrodes were determined from processing of the instant discharge photographs taken with an ICCD camera (see Fig. 5) and summarized in Table 1. The length of the plasma column coincided with the size of the interelectrode gap (plasma column length ~4.5 mm). The diameter of the discharge column was ~0.3 mm and slightly increased with ESU input power. Area of the attachment to the instant cathode was measured from the clearly observed illuminated part of the cathode surface at the moment of maximum discharge current (see images T1 and T2 of Fig. 5). For the replica electrode, this corresponded with the moment *I_d_* peaked during the 1st positive half-wave and occurred at the surgical probe when *I_d_* peaked during the 1st negative half-wave.

**Figure 7:**
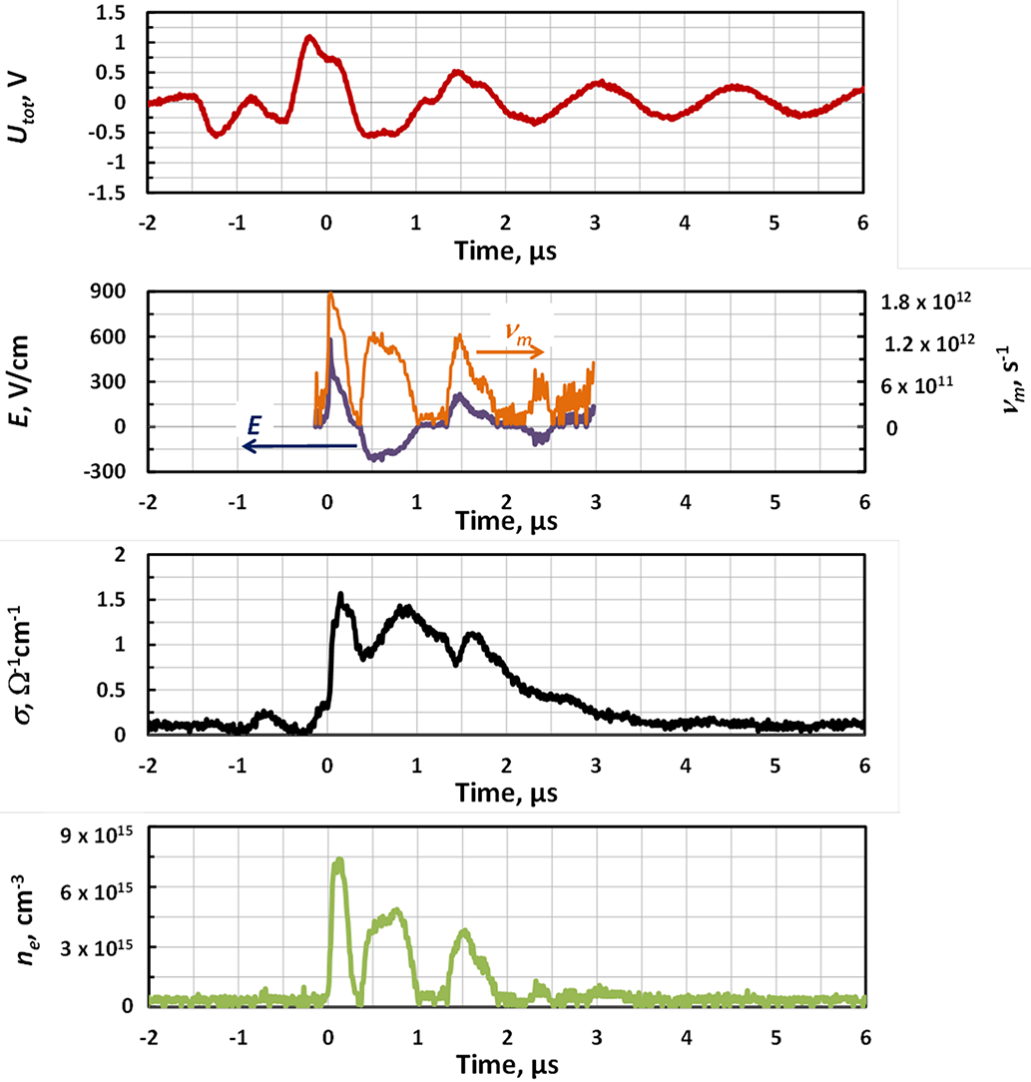
Temporal evolutions of electric field, collisional frequency, plasma conductivity and plasma electron density in the positive column produced by electrosurgical system SS-200E/Argon 2 at *P* = 15 W and argon flow = 3 LPM.

**Figure 8:**
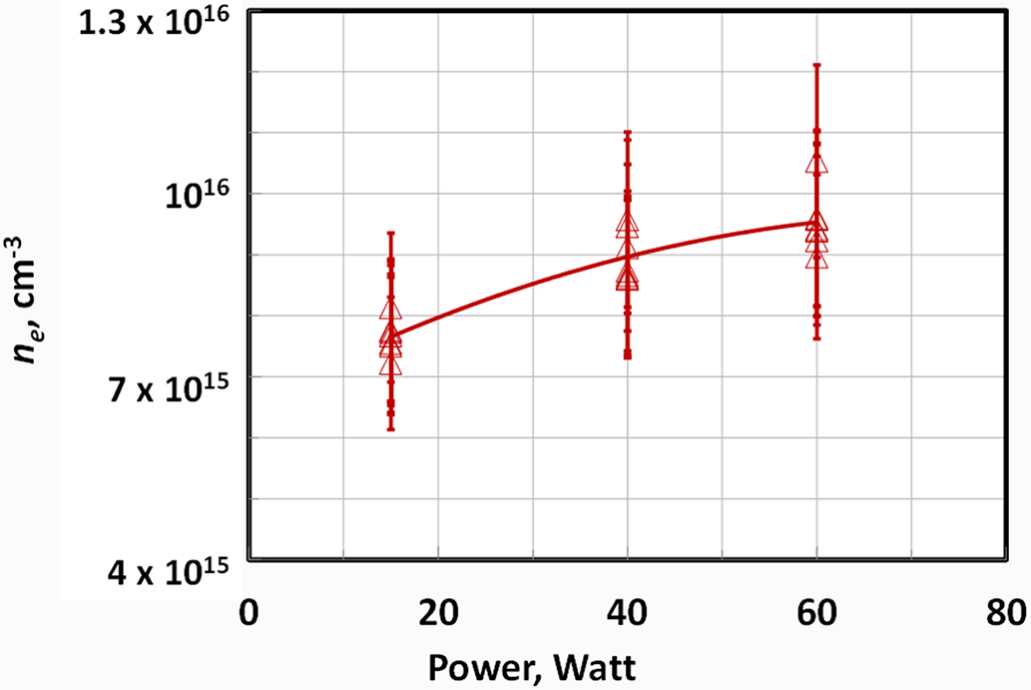
Maximum electron density versus input power of the electrosurgical system SS-200E/Argon 2 (flow = 3 LPM).

Temporal evolution of the average plasma electron density and electric field in the interelectrode column measured using RMS system is shown in Fig. 7. One can see that the peak values of the electric field in the plasma channel and plasma electron density reached about 350 V/cm and 8.10^15^ cm^−3^ respectively, at an ESU input power of 15 Watts. Dependence of the maximum plasma electron density with ESU power is shown in Fig. 8. *n_e_* increased with ESU power reaching about (0.9-1)·10^16^ cm^−3 ^for 60 Watts.

## Discussions

### Temporal behavior

Let us discuss the evolution of the plasma parameters in the positive column and in the near-electrode sheath. The experimental data indicates that the discharge was not continuous during the active stage. Instead, it was interrupted every time the discharge-driving voltage crossed zero (*U_tot_*~0) and was re-ignited on the next voltage half-wave (see Fig. 5 showing that moments when the voltage crosses zero were accompanied by sub-microsecond, quiescent intervals when *I_d_* remained at ~0).

In order to analyze the nature of the discharge interruptions, it is important to discuss plasma decay rates. When discharge driving voltage is changing polarity and the cathode switches to another electrode, the sheath formed near the “old” cathode starts to decay. Photographing of the discharge demonstrates an almost instantaneous termination of the cathode sheath when the discharge driving voltage crosses zero (compare images T1 and T2 in Fig. 5), which can be explained by fast recombination of plasma at the electrode surface^30^. Instant sheath termination causes the discharge current to remain low in the beginning of every next half-wave, until the discharge-driving voltage reaches about *V_br_* ~300 V and breakdown occurs again. The breakdown restores the cathode sheath near the “new” cathode and causes an observed peak in the discharge current (see Fig. 5)^30^,^31^. The discharge lasts until the discharge driving voltage changes sign again and the process is repeated. When the amplitude of *U_tot_* oscillations decreases below 300 V (starting at the 6^th^ half-cycle in Fig. 4), no further breakdown is possible and this indicates the start of the inactive stage, which lasts for about *τ_i_*~12 μs (brighter bar in Fig. 4) when the plasma is decaying.

The positive column also experiences significant decay during the discharge interruptions as one can see from the direct measurements of the interelectrode plasma electron density shown in Fig. 7. Both estimations (using typical electron-ion recombination coefficient ~10^−7 ^cm^3^/s)^30^ and experiments indicate that *n_e_* decays to ~10^14^ cm^−3^ during the discharge interruption lasting about a fraction of a microsecond, when discharge driving voltage is changing sign. This remaining plasma causes a glow in the gap shown in photographs in Fig. 5 during the discharge interruptions in contrast with almost instant termination of the glow around the cathode. It is interesting to note that, in contrast with plasma electron density, the conductivity of the plasma channel does not reduce significantly during the discharge interruptions (see Fig. 7). This is related to the simultaneous decrease of electron collisional frequency around zero *U_tot_* when the electric field in the column is crossing the zero (see Fig. 7).

Therefore, the discharge produced by the electrosurgical system SS-200/Argon 2 represents an AC discharge as opposed to an RF discharge, where near electrode plasma sheaths are permanent and do not decay significantly over the period of oscillation^30^,^31^. The discharge is governed by an instant value of the applied AC voltage and the amount of plasma remaining after the last breakdown event. The discharge interrupts every time when the discharge voltage changes sign due to the decay of the near-cathode sheath and re-ignites on the next voltage half-wave in the vicinity of the “new cathode", when voltage increases above the breakdown threshold and a cathode sheath is formed near the “new cathode”.

### Positive column and near-cathode sheath

**Figure 9:**
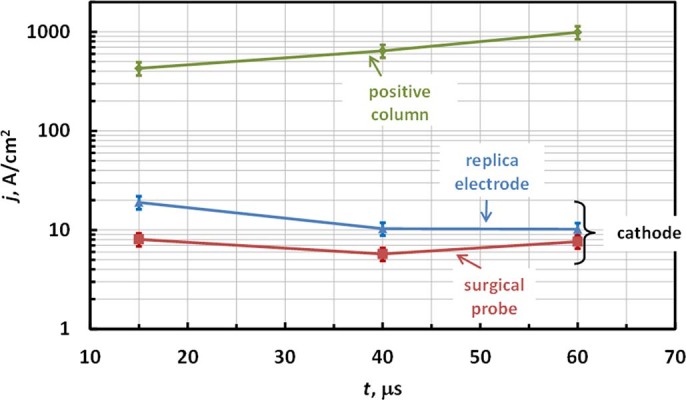
Current densities at cathode and in positive column.

Let us now discuss some parameters of the cathode sheath determined in this work. As shown in Fig. 5, the discharge re-ignites at around *V_br_* ~300 V, which corresponds to a well-known minimum voltage required for self-sustained DC discharges (minimum breakdown voltage of Paschen curve)^30^. The current density at the instant cathode was in the range of 10–20 A/cm^2^ on the stainless steel replica electrode and 5-10 A/cm^2^ on the tungsten surgical probe electrode as show in Fig. 9 (based on measured *I_d_* and area of attachment shown in Table 1). These current densities are close to previously measured normal current densities in glow discharges, indicating that nearly normal cathode layer is established^30^. The depth of the cathode sheath can be now estimated from Paschen curve minimum (using *(p d)_min_*≈1 cm Torr for argon and thus *d_c_*[cm] = 1/p[Torr]) yielding cathode sheath thickness *d_c_*≈10 μm^30^.

It should be noted that higher breakdown voltage ~1 kV observed during the first high voltage oscillation compared to that on the following cycles when breakdown happens around Paschen’s curve minimum ~300 V (see *t*~0 in Fig. 5 and Fig. 6) can be explained as follows. This is caused by the fact that for the first oscillation the entire gap has to breakdown, while on the following half-waves, a well-conducting plasma channel remains after the discharge is interrupted (see conductivity waveform in Fig. 7) and only the near-cathode sheath needs to breakdown.

**Figure 10:**
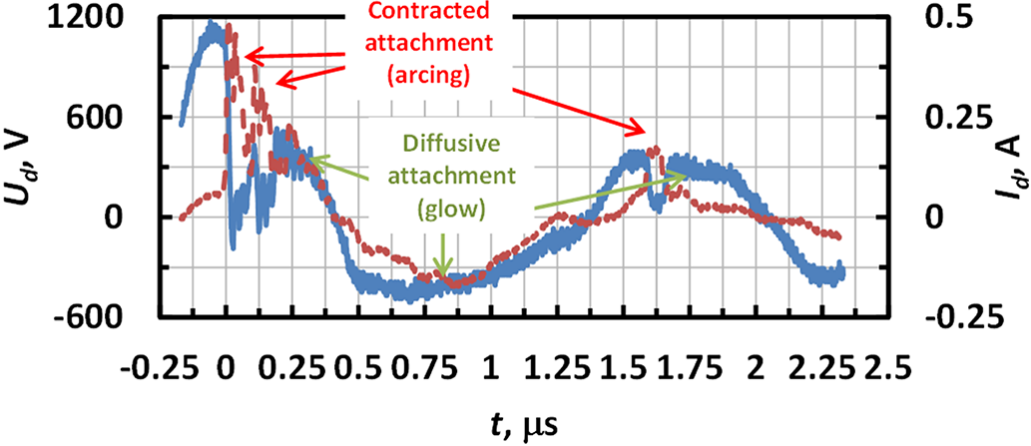
Discharge voltage and current waveforms indicating sporadic transition between modes with steady and non-steady attachment to the cathode on the positive half-wave (ESU power - 20 W, Ar flow rate - 2.5LPM).

Visual observations indicate that attachment of the discharge channel to the living tissue sample may differ from the quasi-steady, diffusive attachment observed at the surgical probe. Instead, the attachment of the plasma column to the tissue was non-steady, accompanied with random motion over the tissue while the plasma column at the point of contact with the tissue was contracted. It should be noted that the scenario of the non-steady contracted attachment can be mimicked with the replica at low ESU powers and flow rates. Visual observations of the attachment to the replica electrode indicate that a decrease of power and flow rate causes sporadic transitions from quasi-steady, diffusive to non-steady, contracted attachment. This transition is captured in Fig. 10 for an ESU power of 20 W and an Ar flow rate 2.5 LPM. It was observed that transition to the non-steady, constricted attachment was accompanied by an abrupt increase of *I_d_* and a decrease of *U_d_* <100V which indicates transition to arc.

Experiments indicate that current density and electrical conductivities in the positive column were up to 10^3^ A/cm^2^ and (1-2) Ohm^−1^cm^−1^, respectively. These values of the current density significantly exceed normal current densities of glow discharges, and along with high measured plasma electron density in the channel *n_e_*~10^16^ cm^−3 ^are typical for contracted (filamented) positive columns^30^. Typically, plasmas at such conditions are nearly thermal and therefore, gas temperatures of about several thousand K might be expected in electrosurgical plasmas^30^. Note, since measured plasma electron density is relatively high, other diagnostic techniques such as Stark broadening and Thomson scattering can be potentially utilized for testing of such plasmas.

Let us now discuss how utilization of the inorganic replica instead of the living tissue affects the discharge. Experiments indicate that discharge attachment was different in these cases, namely non-steady attachment to the living tissue versus quasi-steady attachment to the replica. This can be explained by the fact that application of the ESU to the living tissue causes instant localized burning of the tissue which leads to a change in its local physical properties (electrical conductivity, thermal conductivity etc.) and causes a shift of the discharge column to the new unburnt location. In other words, the tissue can be considered as an electrode with constantly fluctuating properties. Since tissue burning causes carbonization, it may be hypothesized that averaged over time, the surface layer of the living tissue in contact with the plasma would exhibit properties similar to the electrode made of carbon. Also, since the effect of the cathode material on the discharge characteristics (such as cathode fall, breakdown voltage, plasma properties in the positive column etc.) is fairly weak^30^, it can be concluded that the properties of the discharges with the inorganic replica and the living tissue are similar. Similarity of the discharge current and voltage measured with the inorganic replica and with the living tissue also supports this conclusion.

## Conclusions

The discharge produced by the electrosurgical system SS-200E/Argon 2 was studied experimentally. The plasma electron density was measured in the range of (7.5-9.5)·10^15^ cm^−3^ **for applied powers of 15**-60 Watts. The discharge can be classified as a glow discharge of the alternating current with contracted positive column. The discharge ignites every half-wave of the driving voltage when voltage increases above the breakdown threshold and is interrupted at the end of each half-wave when the voltage approaches zero.

## Methods

### Electron density measurements

A methodology for electron density measurement by means of microwave scattering on elongated atmospheric pressure plasma channels will be developed. It should be noted that this consideration differs from that conducted by Shneider in Ref. [23] for short plasma channels when the restoring force produced by the channel ends with non-compensated charges is significant. In contrast, the case for when channels have large aspect ratios (when the contribution of the channel ends is negligible) will be presented. The method described in this section considers the general case of both free-standing plasma channels and those attached to the discharge electrodes. Furthermore, it presents an approach to determine the electric field and takes into account the depolarization effect and dependence of collision frequency on the magnitude of the electric field, in contrast with simplified description in Ref. [22].

Let us consider the scattering of microwaves on the conductive or dielectric channel having slender prolate shape when channel length (*l*) significantly exceeds the diameter (*d*). An incident microwave radiation oriented along the channel excites oscillations of the electric current along the channel. The amplitude of the microwave electric field in the channel for the general case of scatterers with dielectric permittivity ε and conductivity σ when channels are thin compared to skin depth (
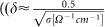
 mm for microwave frequency ~10.58 GHz use in this work) can be written as:
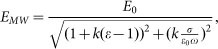
where *k* - depolarization factor governed by the channel geometry, *E*_0_ - incident microwave electric field at the channel location^32^,^33^. The depolarization factor *k* for the channels with large aspect ratio *m = l/d*≫*1* is small: 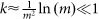
34,35,36.

For the plasma considered in this work, the skin depth *δ∼*0.5 mm (*σ∼*1 Ω^−1^cm^−1^ according to experimental data) exceeds plasma channel diameter *d*~0.2 mm and *m*~20 which yields depolarization factor ~7.10^−3^. Thus, the electric field inside the channel is close to *E*_0_. The amplitude of the resultant electrical current excited in the plasma channel can be written as *I = σ E_0_ S* for a pure conductor and *I = ε_0_(ε−1) ω E_0 _S* for a pure dielectric. The distribution of the current along the channel can be considered nearly uniform if the distance to the radiating horn is large, therefore the front of the incident wave is nearly flat on the channel length: *r_r_*≫*l*^2^*/λ* where *λ* - wavelength of microwave (channel is in a far zone).

Electrical current in the channel oscillates at the frequency of the incident microwave field and therefore, it represents a radiating antenna. In the far-field, when the distance from the detecting horn to the radiating channel *r_d_*≫*l*^2^*/λ*, the radiation pattern coincides with radiation from a Herzt dipole with oscillating current *I*. The overall power radiated by the channel averaged over the microwave period can be written as follows: 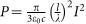
34.

The output signal measured by the linear microwave detection system described in the Experimental Details section is proportional to the electric field in the radiated wave at the location of the detecting horn *E_s_* and thus: 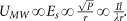. For a microwave system with fixed operational parameters (such frequency, horn locations etc.), the dependence of *U_MW_* is governed by the following propertied of the scatterer channel:


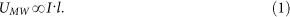


Combining Eq. (1) with the above expressions for current excited in the channel it can be found that the dependence of output RMS signal on parameters of the scattering channel can be expressed as follows:
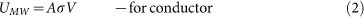

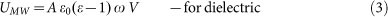
where *A* –proportionality coefficient and *V* - channel volume (*V = S l*).

The proportionality coefficient *A* is a property of the specific microwave system (utilized elements, geometry, microwave power, etc.) while independent of scatterer properties and it can be found using scatterers with known properties. Utilization of prolate-shaped metal cylinders to determine *A* is problematic due to smallness of skin layer depth in metals for microwave frequencies (<1 μm for 10 GHz for copper). Therefore, utilization of dielectric scatterers is preferable to determine *A* using Eq. (3). The constant *A* was found to be 214 V Ω/cm^2^ using dielectric scatterers with known volume and dielectric permittivity similar to that described in details in Ref. [22].

Let us first apply this method to determine the electric field *E* in the tested plasma channel. Note: *E* is assumed here to be governed by the processes developing in the tested plasmas, rather than the amplitude of the microwave electric field in the plasma channel. This imposes a condition on the maximum microwave power level that can be utilized for the diagnostic system to ensure that the entire microwave system is non-invasive with respect to the processes developing in the tested plasmas. The amplitude of the microwave field used in this work can be estimated to be *E_0_* ~ 1 V/cm which is significantly lower than *E* caused by the discharge (see Fig. 7). If the current flowing through the plasma channel is known (e.g. for the case of a discharge initiated between two electrodes), the average electric field in the plasma channel can be determined using Eq. (2) as follows: 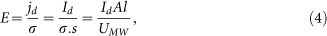
where *j_d_* – discharge current density and *I_d_* –discharge current.

Now let us apply the developed methodology to determine the plasma conductivity and electron density in the plasma channel. For many practical cases, the two following conditions are satisfied for atmospheric plasmas. First, plasma ionization degree is low enough so that electron collisions are governed by collisions with gas particles (*ν_m_*). Second, electron collisional frequency is significantly larger than microwave frequency *ω*, meaning that the plasma channel can be treated as a conductor since the ratio of conductivity current ***j_c_*** to the polarization current ***j_p_*** excited in the plasma channel 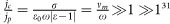 ≫1^31^. Both of these conditions are satisfied for typical atmospheric plasmas (*ν_m_* ~10^12^ compared with *ω*~10^10^, ionization degrees <10^−3^–10^−4^)^30^. In this case, plasma conductivity can be found from Eq (2):
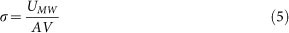


Plasma electron density can be found from σ and electron collision frequency *ν_m_*:^30^


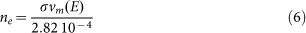


**Figure 11:**
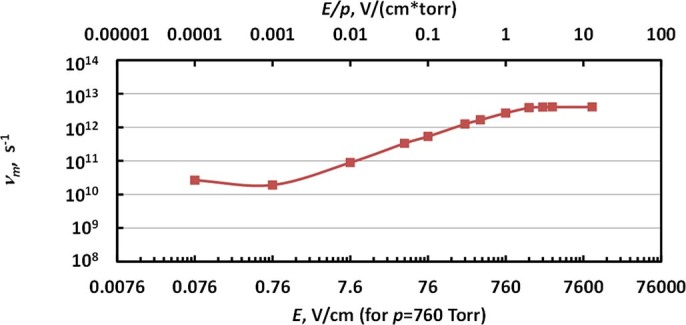
Dependence of electron collision frequency for the case of argon determined for the data published in Ref. [30].

Generally speaking, electron collision frequency depends on the electric field in the channel (*E*) and thus *ν_m_* in Eq. (6) is not constant, but instead *ν_m _ = ν_m_* (*E*). This dependence for argon utilized in this work is plotted in Fig. 11 (determined from data in Ref. [30]). Note, static collisional frequencies shown in Fig. 11 can be utilized if the electric field changes weakly on the time between the collisions (up to frequencies of *E* oscillations <10^10^ GHz). Therefore, the electric field found from Eq. (4) has to be used in combination with dependence *ν_m_ = ν_m_* (*E*) in order to determine the plasma electron density using Eq. (6).

It is important to make a few notes on the applicability of the methodology developed here. First, the method can be applied for both free-standing plasma channels, when radiating antenna size coincides with the size of the plasma channel (such as laser-induced plasma)^25^, as well as for plasmas in contact with the discharge electrodes, when radiating antenna is comprised of plasma column along with pieces of adjacent electrodes. Indeed, since the excitation of the electric current in the channel is driven by the amplitude of the incident wave, microwave scattering from the stationary discharge electrodes contributes to the DC component of the output signal *U_MW_* (similar to the contribution from other surroundings) which is filtered out from the transient signal scattered from the plasma. Second, since it is typical that near electrode sheaths, which form at the contact of the plasma channel with the electrodes, be short compared to the plasma channel length, their contribution to the resultant scattered signal is small. In this case, Eq. (4)-(6) is applicable for measurements of average values of plasma electron density, electrical conductivity and electric field in the positive column.

Summarizing the above spatially-averaged plasma electron density in small atmospheric plasma objects can be determined using scattering of microwaves on the plasmas. In the case of elongated plasma channels, the radiation has to be polarized along the channel and the microwave frequency has to be chosen so that 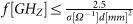 to ensure that channel is thin compared to the skin layer depth. This method measures the total number of electrons in the plasma volume and the current setup has sensitivity down to ~10^11^ electrons. The error of the plasma density measurement is governed by the accuracy of the plasma volume determinations, and it was 10-15% in current experiments. If the electrical current in the plasma channel is measured independently, this method can be also utilized for the determination of the electric field.

## Figures and Tables

**Table 1:** Cross-sectional areas of the positive column and areas of attachment to the instant cathodes. The attachment areas to the instant cathodes were measured at moment of the corresponding discharge current peaks (for the cathode at replica electrode – *I_d_* peak at 1^st^ positive half-wave and for the cathode at the surgical probe - *I_d_* peak at 1^st^ negative half-wave). ESU argon flow rate was set to 3 LPM.

	P = 15W	P = 40W	P = 60W
Cross-sectional area of positive column, cm^2^	7.10^−4^	10^−3^	10^−3^
Area of attachment to the replica electrode, cm^2^	1.6.10^−2^	6.3.10^−2^	10^−1^
Area of attachment to the surgical probe, cm^2^	2.5.10^−2^	6.6.10^−2^	8.10^−2^
