# Erratum: Electric discharge during electrosurgery

**DOI:** 10.1038/srep46955

**Published:** 2018-03-19

**Authors:** Alexey Shashurin, David Scott, Taisen Zhuang, Jerome Canady, Isak I. Beilis, Michael Keidar

Scientific Reports 5: Article number: 9946; 10.1038/srep09946 published online: 04 16 2015; updated: 03 19 2018.

The original PDF version of this Article contained a typographical error in the volume number ‘5’ was incorrectly given as ‘4’. This error has now been corrected in the PDF version; the HTML version was correct from the time of publication.

